# Cellulose-Based Smart Fluids under Applied Electric Fields

**DOI:** 10.3390/ma10091060

**Published:** 2017-09-10

**Authors:** Kisuk Choi, Chun Yan Gao, Jae Do Nam, Hyoung Jin Choi

**Affiliations:** 1 Department of Polymer Science and Engineering, Sungkyunkwan University, Suwon 440-746, Korea; kisuk929@skku.edu (K.C.); jdnam@skku.edu (J.D.N.); 2 Department of Polymer Science and Engineering, Inha University, Incheon 22212, Korea; 22151729@inha.edu

**Keywords:** cellulose, composite, electrorheological, dielectric property

## Abstract

Cellulose particles, their derivatives and composites have special environmentally benign features and are abundant in nature with their various applications. This review paper introduces the essential properties of several types of cellulose and their derivatives obtained from various source materials, and their use in electro-responsive electrorheological (ER) suspensions, which are smart fluid systems that are actively responsive under applied electric fields, while, at zero electric field, ER fluids retain a liquid-like state. Given the actively controllable characteristics of cellulose-based smart ER fluids under an applied electric field regarding their rheological and dielectric properties, they can potentially be applied for various industrial devices including dampers and haptic devices.

## 1. Introduction

In this short review, we deliver smart functionality of celluloses and their derivatives in terms of their electrorheological (ER) response and applications, which has been relatively less well-known to the cellulose community despite their benefits of abundancy and biocompatibility as a raw material and importance of its ER technology. The concepts of eco-efficiency, industrial ecology, sustainability, and green chemistry are leading to a new era in the development of products, materials, and processes. Bio-based polymers produced from biomass feedstocks and renewable agricultural products can be converted into eco-efficient and sustainable products that can compete in the current petroleum-feedstock-based market [1,2]. Among the various natural biopolymers, cellulose is the most abundant organic polymer, and accounts for 1.5 × 10^12^ tons of biomass production annually. It is regarded as an infinite source of raw material to meet the high demand for eco-friendly and biocompatible products [3]. Cellulose can be extracted from variety of agricultural wastes, such as cassava bagasse [4], coconut husk fibers [5], mulberry bark [6], banana rachis [7], wheat, straw, and soy hulls [8,9]. Cellulose contains two phases, the amorphous and crystalline regions at the nanoscale [10,11]. The reduction of the amorphous region can be achieved through acid hydrolysis, which decreases its degree of polymerization [12]. As one of cellulose types, this treated compound is referred to as microcrystalline cellulose (MCC) [13,14], while other types of cellulose such as nanocellulose, cellulose nanocrytals or cellulose nanofibres are also available. Among various applications of celluloses, we mainly focus on the relatively new smart fluid-like characteristics of ER response in this review.

Cellulose particles, along with other types of biopolymers such as chitosan and corn starch, have been utilized as biopolymeric dispersoids for electro-responsive ER fluid systems [15,16,17,18] ever since its first discovery in ER fluids due to not only their sustainability/green/environmental perspective, but also the structure morphological perspective that highly crystalline microparticles and cellulose derivatives (with different degree of substitution) can exhibit. Note that the ER fluids are well known as electro-responsive smart and intelligent materials. They normally consist of electrically polarizable sub-micron to micron-sized particles in insulating fluids, in which the dispersed particles can be polarized in the presence of an external electric field. Thus, they have a unique ability to form fibrillar structures under applied electric fields [19,20,21] by undergoing transient aggregation into a solid phase because of the attractive forces between the dipole moments of the dispersed electro-active particles. These field-induced dipoles come in contact with each other and create chains or fibrillated structures along the electric field that are strong enough to restrain fluid flow. Separate chains can also be attracted to each other, forming a multi-structured chain through inter-chain forces. Thus, ER suspensions exhibit phase transformation from a liquid-like to a solid-like state under different electric field strengths, and the transition is reversible and very rapid when the external field is removed [22], resulting in the changes of rheological properties including shear stress, mechanical modulus, and shear viscosity of the ER fluids. Because of the easily controllable phase transition characteristics of these materials, smart and intelligent ER materials have drawn great attention for their industrial applications, such as damping systems, brakes, ER polishing, and haptic devices in addition to the automotive industries and military systems [23,24,25,26,27]. Concurrently, to overcome the drawbacks of hydrous ER materials, which include water evaporation, thermal instability, and device corrosion, various anhydrous ER systems have been studied [28]. Thereby, cellulose and its family have been introduced as an ER material since the beginning of ER research and development.

This review covers the overall insight into the behavior of smart fluids based on cellulose and its derivatives among many other potential ER materials. Typical properties of cellulose are briefly explained and then several behaviors of ER fluids based on cellulose and its derivatives, such as their chemical, morphological, dielectric and rheological properties are discussed, along with the principles and characteristics of conventional ER fluids. Various ER fluids based on pristine cellulose and its derivatives and composites, such as MCC, phosphate cellulose, titania/hydroxypropyl cellulose, and cellulose/carbamate are followed, as well as their hydrous and anhydrous systems.

## 2. Cellulose

The biopolymeric cellulose is regarded as the most abundant natural polymer on earth, and the most common lignocellulosic material can be found in tree wood. Other cellulose containing materials include water plants, grasses, agricultural residues, and other plant materials. Plants are composed of not only cellulose, but also lignin, hemicellulose, and other extractives, while commercial cellulose production is based on agricultural wastes, such as highly cellulosic based cotton materials. Table 1 lists the constituents of various natural sources [29], highlighting the availability of cellulose from abundant natural resources. Not only can cellulose be easily obtained from plentiful natural resources, but it also possesses desirable properties such as swelling, chemical stability, and toxic resistance under harsh temperature and pH [30].

**Figure 1:**
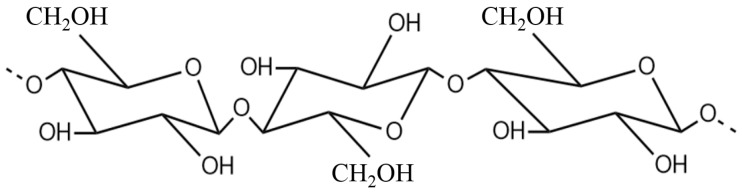
Chemical structure of cellulose.

Cellulose is a polysaccharide linear chain that contains hundreds to thousands of β-1, 4 linked D-glucose units, as shown in Figure 1. Many hydrogen bonds are formed between oxygen and hydrogen inter- and intra-molecularly because of the multiple hydroxyl groups on cellulose. As a result of these forces, the cellulose chains join to form micro-fibrils which lead to a very high tensile strength [31] and elastic modulus of 10 and 150 GPa, respectively, for pure crystalline cellulose [32]. In addition, a high Young’s modulus of 128 GPa is observed in the case of plant cellulose fibers [33]. Cellulose with a low density also shows good damping performance due to its hollow fiber surroundings.

Furthermore, the reactive surface of cellulose can undergo modification to form composites; among various applications, cellulose has been widely used as a biodegradable natural filler in polymer composites. Nonetheless, despite the above advantages of cellulose, reinforced composites with natural fibers still require a great deal of improvement. Cellulose, because of its polarity and hydrophilicity, is poorly compatible with hydrophobic and nonpolar thermoplastics, which results in poor dispersion of fillers and weak interaction between matrices and fillers. Cellulose fiber composites can also result in poor mechanical properties due to swelling. This illustrates that anhydrous materials are preferable as fillers for composites [31]. Nonetheless, this hydrophilic behavior of cellulose renders its potential usage as ER materials.

Cellulose, which is derived from different plants and microorganisms exhibits different characteristics [3]. For example, rice husk cellulose is utilized in various applications, such as animal husbandry, pest control, and absorbents [34], while rice husk ash powder produced by combustion processes has been adopted for various applications such as steel, refractory bricks, and partial cement replacements as an additional filler [35].

Furthermore, the promising potential applications of cellulose have led to the development of new derivatives, such as cellulose acetate, carboxylmethyl cellulose, and phosphate cellulose. Phosphate cellulose was originally introduced to replace cellulose-based flame retardant textiles for fireproof materials [36]. The phosphate functional group can also be applied for broad biological binding to create specialized active surfaces [37,38], as well as having ion exchange ability for applications related to calcium-related diseases [39]. MCC has been used in a variety of applications such as the medical, cosmetics, and food industries as a flow characteristics controller, suspension stabilizer, and ion exchanger [40] in gel formations to increase the quality of the final products [41]. Many studies have been conducted on biodegradable bio-composites and the development of methods to improve their biodegradability and mechanical properties. These properties also enable their use in a wide range of applications which require high stiffness and strength, including cardiac devices and biodegradable bags [42].

**Figure 2:**
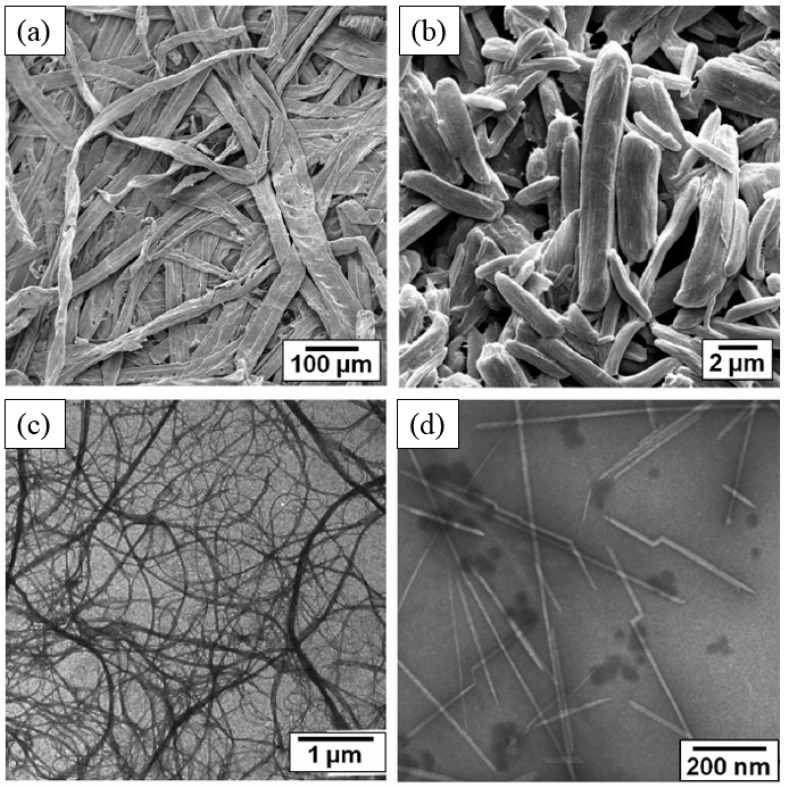
SEM images of different cellulose particle types: (**a**) wood fiber; (**b**) MCC; (**c**) microfibrillated cellulose; and (**d**) tunicate cellulose nanocrystals (adopted from [1]).

Among various types of cellulose, particulates are appropriate for their ER application and it is well-known that various types of cellulose particles have been reported from different source materials and extraction processes, including wood fiber, MCC, micro-fibrillated cellulose, tunicate cellulose nanocrystals, and others. Figure 2a observed from Scanning Electron Microscopy (SEM) shows the largest size (length > 2000 μm) cellulose particles, wood fiber particles, which play an important role in the paper, textile, and biological fields. Generally, wood fiber particles have a special hierarchical structure and a comparatively low crystallinity (43–65%) [1]. The rod-like MCC particles have a length of about 1–10 μm, and are porous materials having a wide range of applications in the drug and food industries. They have a higher crystallinity of 80–85%, as shown in Figure 2b. Micro-fibrillated cellulose materials are prepared by refining highly purified wood fiber and plant fiber paper pulps. They have multiple elementary fibrils at their fiber ends. In addition, they have a high aspect ratio and almost complete cellulose containing amorphous and crystalline structures, as depicted in Figure 2c. Figure 2d shows the ribbon-like tunicate cellulose nanocrystals derived from the acid-hydrolysis of tunicates. Nano-sized tunicate cellulose nanocrystal particles have a high aspect ratio of about 70–100 (length: 100–4000 nm; width: ~20 nm; height: ~8 nm) and an almost complete crystallinity of 85–100%; their crystallinity and aspect ratio are the highest among cellulose nanocrystal particles [1].

**Figure 3:**
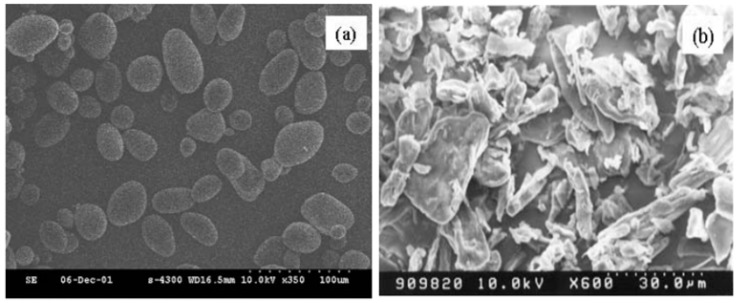
SEM micrograph of: (**a**) potato starch phosphate; and (**b**) cellulose phosphate particles [43].

On the other hand, Figure 3 shows SEM images of both polysaccharide potato starch phosphate (PSP) particles and cellulose phosphate (CP) particles synthesized using an esterification process with urea and a mixture of ortho-phosphoric acid at atmospheric temperature, which was detailed in a previous study [43] for comparison. The cellulose phosphate particles showed irregular and rod-like shapes compared to the round-shaped potato starch phosphate particles even though both belong to same polysaccharide phosphate family. The morphology of the cellulose phosphate particles did not change after phosphorylation, with their size ranging 10–50 microns.

**Figure 4:**
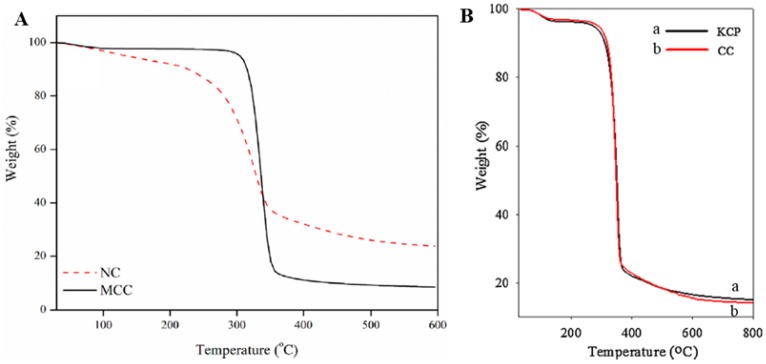
(**A**) TGA curves of MCC and nanocellulose (NC) (reproduced from [45]); and (**B**) TGA curves of kenaf core pulp and cellulose carbamate (reproduced from [46]).

The thermal properties of the dispersed particles in ER fluids are important because of the potential engineering applications for ER fluids in a wide range of temperatures [44]. Concerning this issue, the thermal decomposition properties of MCC and nanocellulose were tested using thermal gravimetric analysis (TGA) under a protective nitrogen atmosphere, with a heating rate of 10 °C/min up to 600 °C, as shown in Figure 4a [45]. In the case of MCC, a small amount of degradation (2%) at around 100 °C was attributed to the absorbed water. The next degradation was detected between 300 and 370 °C, and was mainly due to the formation of alkenes and other hydrocarbons in the cellulosic materials [45]. For nanocellulose, the main weight loss was observed at a lower temperature range, from 220 to 340 °C, which may be ascribed to amorphous matter remaining in the nanocellulose. The TGA data show that the nanocellulose had better thermal stability (77% degradation) than the MCC (91% degradation).

Figure 4B shows the TGA curves of kenaf core pulp and its derivative, cellulose carbamate. Their decomposition stages were very similar. The first stage was related to weight loss from bonded or absorbed water around 100 °C. The second stage occurred from 300 to 400 °C, and was due to the degradation of the kenaf core pulp and cellulose carbamate; their weight residues were 15.07% and 14.19%, respectively. The weight of the undecomposed kenaf core pulp was slightly higher than that of cellulose carbamate, which was attributed to ash in the kenaf core pulp as a result of its being employed without a purification process [46].

## 3. Electrorheological Fluids

**Figure 5:**
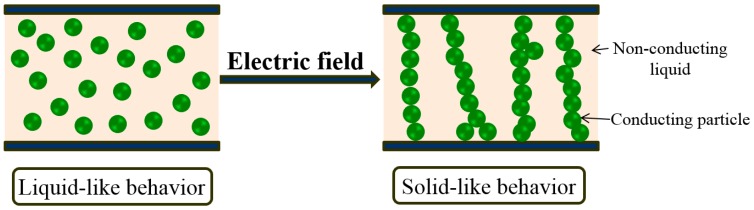
Schematic diagram depicting phase transformation behavior of ER fluid under an applied electric field.

ER fluids are one of the most interesting smart materials. They are suspension systems of dielectric/semi-conducting particles dispersed in nonpolar liquids, which construct fibrillated particle structures under applied external electric fields due to the difference in dielectric constant between the particles and the insulating oil [47], as shown in Figure 5. In contrast, ER gels and ER elastomers have either an elastomer or gel suspension medium, respectively [48]. Among the various polarization mechanisms, interfacial polarization is known to be closely related to ER phenomena [49,50]. The reversible phase transition from liquid-like to solid-like provides control of a variety of viscoelastic characteristics such as yield stress, shear viscosity, and dynamic modulus by using applied electric fields [51,52]. Because of this, ER fluids have drawn great attention as smart functional materials [53]. Most ER fluids show Newtonian-like liquid behavior, with a slope of 1.0 when shear stress is plotted against shear rate in log-log graph. However, an applied electric field induces the transition of the liquid-like state into a solid-like state through the formation of a column like structure along the electric field direction. The competition between the hydrodynamic breakdown under an applied shear during rheological measurement and the attractive force under an applied electrical field is responsible for many interesting rheological characteristics of the ER fluids. Because of this, the rheological behaviors of ER fluids have been described using various rheological equations of state, including the Herschel–Bulkley fluid model, Bingham fluid model, [54], Seo–Seo model [55], and Cho–Choi–Jhon model for flow curve analysis [56].

Many early ER systems including cellulose were based on hydrous particles [57]. To overcome the disadvantages of hydrous ER systems such as corrosion and thermal instability, various anhydrous particles have been used. These include various conducting polymers such as copolyaniline [58,59], polyaniline, polypyrrole [60], poly(acene quinone) radical [54], carbonaceous materials, polyindole, [61] and their inorganic hybrids [62]. The polymers with π-electron systems show unique electronic properties, such as low ionization potentials and high electron affinities [28]. New anhydrous biopolymers such as potato starch phosphate [63], chitosan, [64,65], and cellulose phosphate [66] have also been incorporated in ER fluid systems. Note that water is very important for dielectric polarization in hydrous cellulose, while the substitution of phosphate plays a crucial role in anhydrous phosphate systems. The field-induced structure is generated in such a way that original charge carriers in surface or bulk of particles flow with applied electric field [67,68,69]. The ER performance of phosphoric ester phosphate cellulose has been studied [15,70], in which phosphate cellulose was synthesized by an acid-urea mixture esterification between cellulose and phosphoric acid [66].

Three distinctive types of ER fluids have been developed: (1) positive ER fluids; (2) negative ER fluids; and (3) photo-ER fluid. Most ER fluids that have rheological properties, such as shear stress and viscosity, which increase under an applied electrical field, belong to positive ER fluids [71,72,73]. In contrast, ER fluids that have the opposite behavior (i.e., those that show decreased shear stress and viscosity under an applied electrical field) are called negative ER fluids [74,75,76,77]. However, the characteristics of both types of ER fluids can be improved through light lamination; these are called photo-electrorheological (photo ER) fluids [78,79]. Photogenerated carriers are considered to be responsible for changing the electric properties of the positive or negative materials and enhancing their ER phenomena. The alignment of ER particles is observed in positive ER fluids, and existence of fibrils becomes apparent as the electric field increases [80,81]. Additionally, two related phenomena have been also discovered: Quincke rotation (electro-rotation) and phase separation (electro-migration). Quincke rotation is the steady electro-rotation of an insulated particle suspended in a conducting fluid under a high electric field [82,83].

ER fluids have been used in various engineering applications such as e-ink, brakes, mechanical sensors, human muscle simulators, haptic services, damping systems, and polishing media [84,85,86], taking advantage of the ability to control their mechanical properties under different electric field strengths. Furthermore, the fact that the change in viscosity behavior sustains Newtonian fluid characteristics is a noteworthy advantage of negative ER fluids [87,88].

## 4. Cellulose Based ER Fluids

The plentiful biopolymer cellulose is an environmentally friendly green material which shows outstanding characteristics and a wide variety of applications, which makes it an understandably appealing research topic [89,90]. Its semi-conducting properties, which are due to a small amount of adsorbed water molecules, mean that it is a unique functional material. The ER properties of water activated cellulose under applied electric fields, have been extensively investigated [18], particularly in terms of the role of water. Stangroom [91] reported that mobile ions in the particle pores assembled with water molecules to serve as bridges under an electric field. Stipanovic and Schoonmaker [92] proposed that water molecules adsorbed in the crystal lattice of hydrated polysaccharide chains could promote ER performance. Zhang et al. [18] considered the absorbed water as unfrozen bound water, and showed that the ER performance of water based cellulose rose as the water content increased up to 8.5% *w*/*w*, and then decreased. The critical moisture content was close to the transformation of the slightly mobile “liquid-like” unfrozen bound water to the highly mobile “liquid-like” unfrozen bound water. For a fixed moisture content, the yield stress of ER fluids improved gradually when either the amount of cellulose or the electric field strength was increased. However, the ER behavior declined when the temperature was raised.

MCC is a depolymerized cellulose obtained from fibrous-plant-derived alpha cellulose, and is known to possess a higher specific area compared to typical celluloses. MCC has also been incorporated in ER materials. Sim et al. [93] reported a MCC particle based ER fluid which was obtained from natural pristine rice husk in a three stage process. Alkali treatment was the main process to remove hemicellulose from the milled pristine rice husk; a bleaching process was then used to obtain a more refined cellulose material; and, as the last step, hydrolysis with sulfuric acid was used to break down the amorphous region to form the MCC particles [93]. In general, plant fibrils contain two domains, the crystalline and amorphous regions. MCC can be acquired by breaking down the amorphous region using acid hydrolysis [94]. The micro-sized cellulose exhibits a higher specific surface area than traditional cellulose; thus, MCC particles have higher mechanical properties and thermo-stability values due to the strong interactions within particles [95,96].

The ER performance of MCC in different oils under a low external electric field has been reported. Davies et al. [97] reported that MCC in super refined BP oils generally exhibited higher yield stresses than in conventional silicone oil in the absence of an electric field and under a low applied electric field of 500 V/mm. Almond oil showed no obvious differences from silicone oil, whereas peanut oil, sesame seed oils, and safflower oil showed higher values than silicone oil, which was attributed to the oleic acid in BP oils. It has also been demonstrated that MCC particles demonstrate ER effects in pharmaceutically acceptable oils under a low electric field, which may be useful for practical applications. On the other hand, rod-like commercial MCC was suspended in silicone oil and exhibited an ER effect [98], in which the shear stress was proportional to the weight fraction of cellulose. This was interpreted to mean that the number of particle chains were proportional to the weight fraction. The yield stress also was proportional to the weight fraction, indicating that the polymer suspension did not form a gel at high cellulose particle weight fractions. While the shear stress was proportional to the square of the voltage, the corresponding current had a relationship of the fourth power of the applied voltage due to increased formation of the cellulose chains with increasing applied voltage.

In addition, it can be also noted that even though other types of cellulose such as nanocellulose, cellulose nanocrystals or cellulose nanofibres were examined in many different aspects, they have never adopted as ER materials.

## 5. Cellulose Derivatives and Composite Based ER Fluids

In addition to many ER studies based on natural celluloses, which are mainly hydrous systems, the use of cellulose derivatives and cellulose composites as anhydrous ER materials has also been investigated. Tilki et al. [17] reported typical ER properties for an ER fluid based on modified cellulose (MC) which had been initially transformed into a carboxyl salt. They examined the ER behaviors of both pure cellulose and MC particles dispersed in corn oil. In the absence of an electric field, the viscosity decreased and the shear stress increased with increasing shear rate, showing a typical Newtonian fluid behavior, and the shear viscosity and shear stress of the cellulose based ER fluid were higher than those of the MC system. However, in the presence of an electric field, the MC based ER fluid exhibited a greater ER response than the cellulose based system due to the stronger induced polarization forces of the MC. In addition, Bingham plastic behavior was observed under the applied electric field. In another study, measurements of the ER effect and dielectric characteristics of a hydroxypropyl cellulose suspension, in which the cellulose particles contained 2 wt % water, were conducted at the same time [99]. In the absence of an electric field, the fluid showed Newtonian behavior, with shear stress proportional to the shear rate. A different phenomenon was observed when ac and dc fields were applied. Under a dc field, the shear stress increased with increasing shear rate, while the relationship changed dramatically according to the frequency under an ac field. The dielectric measurement demonstrated a nonlinear relationship in the low frequency range, containing a small elevation of the shape of the peak.

Furthermore, the fabrication of cellulose composites is a novel method to enhance the performance of cellulose, especially its electrical conductivity [100]. Kraev et al. [101] synthesized titania/hydroxypropyl cellulose particles via a sol-gel process and observed an enhanced ER effect. Composites which possess the chemical and electric characters of polyaniline (PANI) and the flexibility, availability, and suitable surface areas of cellulose materials have also been fabricated [102]. Compared with bilayer PANI/cellophane actuators, the trilayer PANI/cellophane/PANI actuators were found to display higher actuation parameters. Poly(ethylene glycol) (PEG)/cellulose blends were also manufactured by mixing cellulose xanthate solution and PEG solution in different ratios [103]. The dielectric constant (ε) of the blends declined with the increasing frequency due to dielectric dispersion, and an increasing trend for ε was observed with both increasing PEG content and increasing temperature up to 80 °C. The value of ε then decreased as the temperature was increased to 100 °C, which was attributed to the phase transition [104].

As biodegradable materials, cellulose films are compatible with in vivo applications [105]. A self-propelled drug releasing system was developed, in which cellulose/polypyrrole (cellulose/PPy) composite films were coated with an active metal on one side. The drug contained in the PPy films could be delivered effectively in the electrolyte solution. The efficiency of the drug delivery could be tuned by varying the type and thickness of the coated metals [106]. Recently, a composite based on cellulose carbamate (CC), which is an environmentally friendly material due to its biodegradability [107], was synthesized from kenaf core pulp by a microwave irradiation process [46] and used in an ER suspension.

## 6. ER Characteristics of Cellulose and Its Derivative Materials

The rheological characteristics of cellulose-based ER fluids, including the yield stress, flow curve of shear stress, shear viscosity, and dynamic moduli under steady and dynamic shear modes are considered to be important parameters for their development.

**Figure 6:**
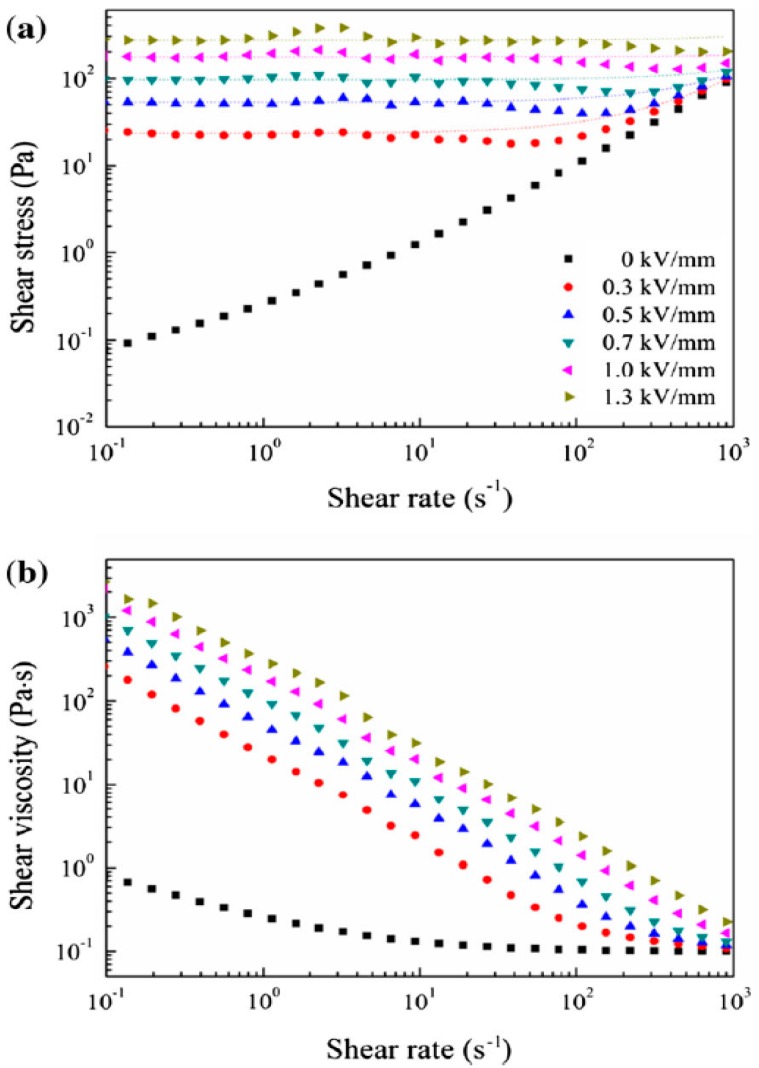
(**a**) Shear stress vs. shear rate; and (**b**) shear viscosity vs. shear rate of MCC ER fluid under a series of external electric field strengths. The dotted line in (**a**) is calculated from the Bingham equation (reproduced from [93]).

Figure 6a shows a plot of the shear stress versus the shear rate of the MCC ER fluid, in which the MCC particles were triturated meticulously and dispersed in silicone oil at 10% *w*/*w*. The ER performance of the MCC based ER fluid was studied using a rotational rheometer under a series of external electric field strengths. In the absence of an external electric field, the MCC ER fluid displayed a Newtonian fluid-like behavior, with the shear stress increasing almost linearly with increasing shear rate. Upon the application of the external electric field, the yield stresses were observed as soon as shear was applied and remained nearly constant, indicating that the ER fluid exhibited non-Newtonian fluid properties. As the electric field strength was raised, the yield stress increased gradually, since the ER fluid formed stronger chain-like structures parallel to the electric field direction. The Bingham fluid model is commonly used as the simplest model for ER fluids, and is given as Equation (1) below:

(1) $$\tau =\tau_{0}+\eta \dot{\gamma }\left(\tau \geq \tau_{0}\right)\;\dot{\gamma }=0\;(\tau <\tau_{0})$$

where τ represents the shear stress, τ_0_ stands for the yield stress, η is the shear viscosity, and $\dot{\gamma }$ denotes the shear rate. The dotted lines seen in Figure 6a were generated by fitting the flow curves of the MCC ER fluid to Equation (1); the optimized parameter values are listed in Table 2.

In addition to the shear stress data, the connection between the shear viscosity and shear rate is depicted in Figure 6b. Similar to the shear stress data, in the absence of an external electric field, the ER fluid demonstrated Newtonian fluid behavior with liquid-like structures, showing a nearly constant shear viscosity. Whereas, on the basis of the relatively lower disperse state or higher concentration, ER fluids display imperfect Newtonian properties in the absence of an electric field [108]. Under an applied external electric field, a substantially increased shear viscosity was observed due to the orderly chain-like structures induced along the electric field direction. The shear viscosity declined with increasing shear rate, demonstrating showed typical shear thinning behavior, which resulted from the oriented chain structure being warped or destroyed.

**Figure 7:**
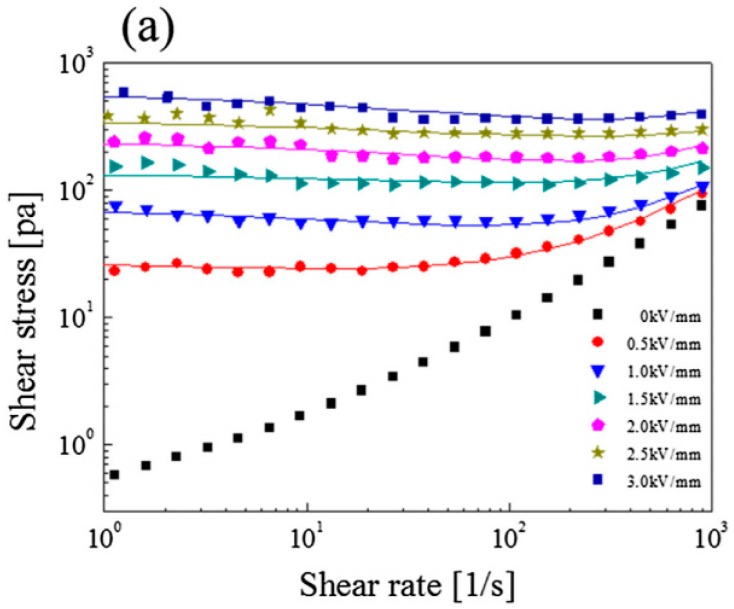

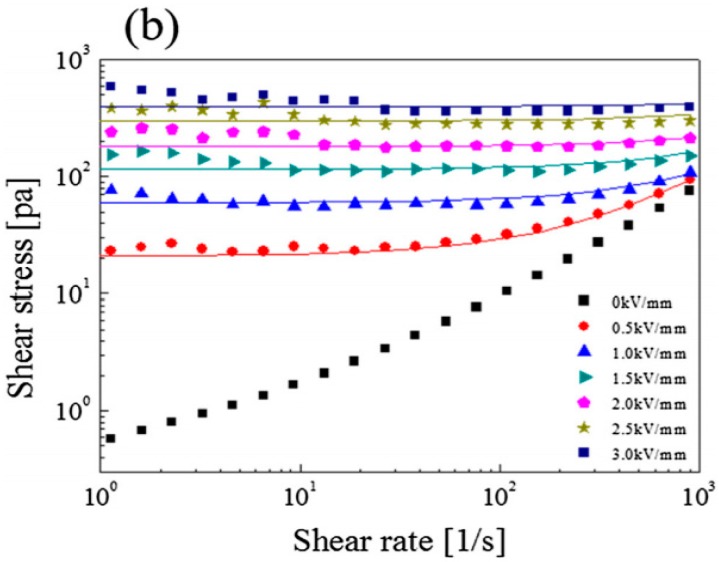
Flow curves of the stress of the phosphate MCC ER fluid under different electric field strengths: (**a**) fitted by the Cho–Cho–Jhon (CCJ) model; and (**b**) fitted by the Bingham fluid model [70].

The ER behavior of anhydrous phosphate cellulose was also analyzed. The ER characterization of 10% *v*/*v* phosphate MCC particles dispersed in silicone oil was conducted using a controlled shear rate (CSR) test under a range of electric field strengths. As shown in Figure 7, the phosphate MCC particle based ER fluid showed Newtonian fluid behavior in the absence of an electric field, with the shear stress increasing proportionally with the shear rate. However, non-Newtonian behavior was observed for the phosphate cellulose based ER fluid when an electric field was applied. In other words, yield stress was observed at a shear rate of approximately zero, and the shear stress increased with increasing electric field strength. The shear stress also showed a plateau, becoming more constant at higher electric field values, and became stable beyond 2.5 kV/mm. This occurred due to polarization; the polarized particles create attractive forces and form fibril structures. This structure can maintain the flow characteristics until the shear rate reaches a maximum value at which the fibril structure breaks [109]. In comparison to hydrous MCC fluids, anhydrous phosphate MCC exhibits lower yield stress in the same electric field range [93].

The flow curves were fitted using both the Cho–Cho–Jhon (CCJ) and Bingham equations [110]. Both the CCJ model and the Bingham fluid model were used to compare the data from both models, and to prove that the CCJ model fits the complex data better. The CCJ equation is given in Equation (2), and the Bingham fluid model in Equation (1).

(2) $$\tau =\frac{\tau_{y}}{1+{\left(t_{1}\cdot \dot{\gamma }\right)}^{a}}+\eta_{\infty }\left(1+\frac{1}{{\left(t_{2}\dot{\gamma }\right)}^{\beta }}\right)\dot{\gamma }$$

Equation (2) includes six parameters that have been used to fit the experimental values for a range of various ER fluids. $\tau_{y}$ represents dynamic yield stress, which is extrapolated from the shear stress from low shear rate, $\eta_{\infty }$ is the shear viscosity at a high shear rate, and *t* is the time constant. The exponent a is related to the decrease of shear stress and *β* is in the range of 0 < *β* ≤ 1 as dτ/d $\dot{\gamma }$ ≥ 0 [111]. Equation (2) was used to produce the solid lines shown in Figure 7a, and the CCJ model fit well in both the high and low shear rate regions for the phosphate MCC based ER fluid. The descriptions of the symbols are as follows. First of all, τ is the shear stress value, $\tau_{y}$ relates to the volume fraction, particle, external field strength and $\eta_{0}$ represents the viscosity of the ER fluid at a high shear rate [112]. The CCJ model describes the general shear thinning behavior due to the formation of a fibrillate structure at various electric field strengths. The reforming phenomenon was examined in the low shear rate region, as the electrostatic interactions are stronger than the hydrodynamic breaking force, which leads to higher shear viscosity this region. This proves the shear thinning behavior more clearly [70].

**Figure 8:**
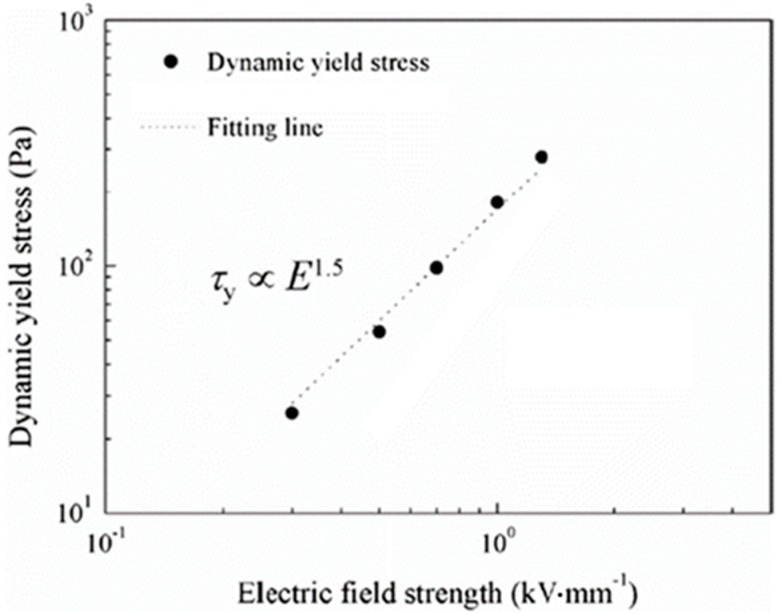
Dynamic yield stress vs. electric field strength for the MCC ER fluid under a series of external electric field strengths. The dotted line in the figure was obtained from the expression $\tau_{y}\propto E^{1.5}$ (reproduced from [93]).

To intensively study the ER properties of the MCC based ER fluid, the $\tau_{y}$ data in Figure 6a were re-plotted. The double logarithmic coordinates of dynamic yield stress ($\tau_{y}$) versus electric field (E) are indicated in Figure 8, and the dotted line is a fitting line, in which the ${\;\tau }_{y}$ values of the MCC ER suspension and E were related by the formula of $\tau_{y}\propto E^{n}$. Here, the exponent is 1.5, in accordance with the conduction model [113,114,115].

**Figure 9:**
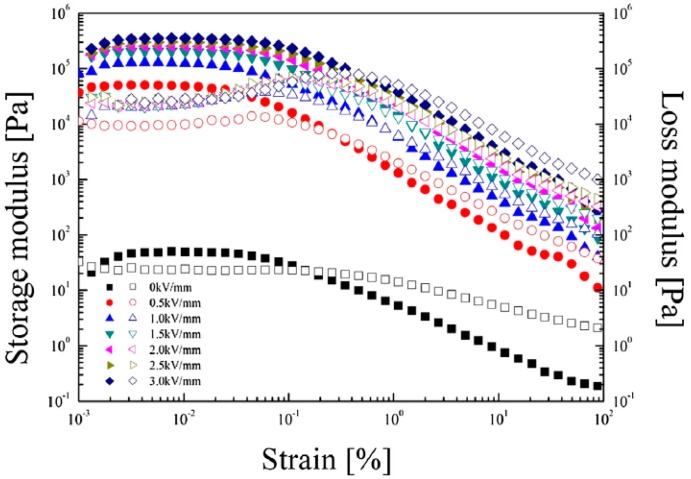
Storage modulus (filled symbols) vs. strain and loss modulus (open symbols) vs. strain for the phosphate MCC ER fluid under a series of external electric field strengths (reprinted from [70]).

The dynamic properties of the phosphate MCC based ER fluid were measured in an oscillatory experiment to determine its viscoelastic behavior. The system was fabricated by adding 10% *v*/*v* of phosphate MCC particles to a silicone oil under ultrasonication to obtain a dispersion. As shown in Figure 9, the amplitude sweep test was carried out at a set frequency of 6.28 rad/s in the strain range from 0.001 to 100%. In the linear viscoelastic region of strain ($\gamma_{\mathrm{LVEr}}$), the storage modulus (G’) was higher than the loss modulus (G”) whether the electric field was absent or present, showing that this system demonstrated obvious elasticity. The $\gamma_{\mathrm{LVEr}}$ broadened with increasing electric field, which was attributed to the enhanced inter-particle polarization forces of the ER fluid. However, as the strain was increased, critical strain points at which G’ was equal to G” were observed. In the high strain region after the critical points, G” was greater than G’ due to the viscosity of the fluid. Thus, the speed of the deformation of the chain-like structures was higher than the speed of their formation whether or not an electric field was applied.

**Figure 10:**
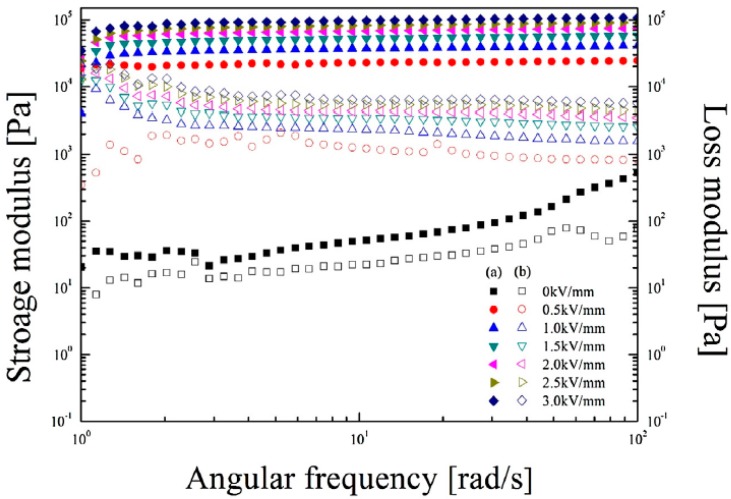
Storage modulus (filled symbols) vs. angular frequency and loss modulus (open symbols) vs. angular frequency for the phosphate MCC ER fluid under a series of external electric field strengths (reprinted from [70]).

A strain value of 0.005% was selected from within the $\gamma_{\mathrm{LVEr}}$ to perform a frequency sweep test of the phosphate MCC ER fluid under a series of different electric field strengths, as shown in Figure 10. The angular frequency was varied from 1 to 100 rad/s during the test. In the absence of an electric field, the values of G’ and G” increased obviously along with increasing angular frequency, showing a liquid-like property. In the presence of an electric field, G’ and G” were approximately constant throughout the entire angular frequency region, proving that this suspension was in a solid-like state. Compared to G”, G’ showed higher values throughout the whole frequency region, under different applied electric fields or zero electric field. Thus, the phosphate MCC ER fluid is dominated by elasticity characteristics rather than viscous properties.

**Figure 11:**
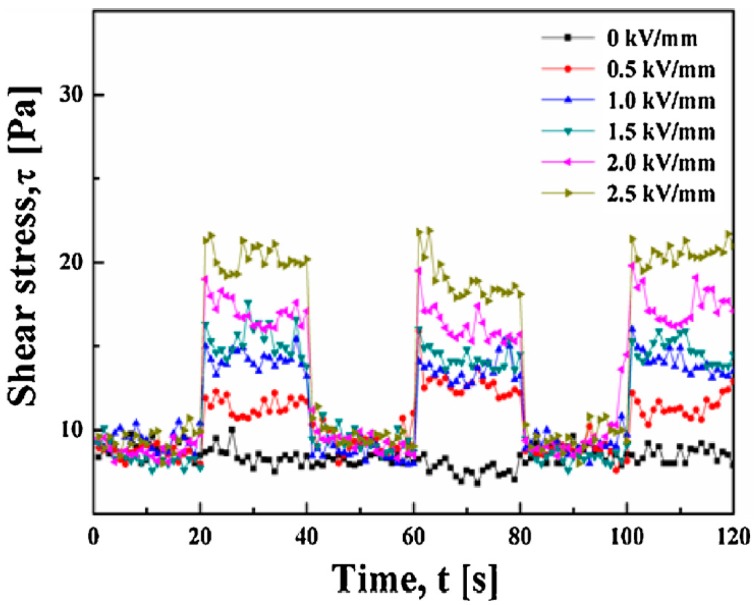
Shear stress vs. time at a set shear rate (1/s) for a 0.5 vol % cellulose carbamate (CC) based ER fluid under a series of external electric field strengths (reprinted from [46]).

Figure 11 displays the steadiness and sensitivity of the CC material based ER fluid at a set shear rate of 1/s and a square-wave pulsed voltage signal of 20 s. During each cycle, the electric field was turned on and maintained at a constant voltage for 20 s, then switched off for 20 s. Data were plotted every 0.1 s. When the electric field was applied, the shear stress increased to a higher value, which remained relatively stable until the electric field was turned off. In addition, the shear stress jumped to the higher value over 0.1 s, since the off–on (or on–off) borders contained few points. Namely, the CC particles needed little time to form and strengthen the chain-like structures under an applied electric field. At the off–on (or on–off) borders, the first datum usually remained unchanged from the previous value, indicating that the response time ranged 0.1–0.2 s [116]. In addition, the shear stress increased with the increased voltage when the field was switched on.

**Figure 12:**
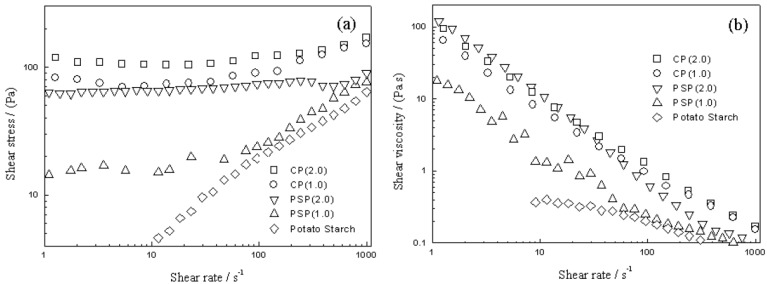
(**a**) Shear stress and shear rate of PSP (1.0 M), PSP (2.0 M), CP (1.0 M), and CP (2.0 M), and potato starch; and (**b**) shear viscosity and shear rate of PSP(1.0 M), PSP (2.0 M), and potato starch [43].

As shown in Figure 12, CP shows higher shear stress than PSP, which might be due to cellulose having a higher conversion to phosphate than potato starch, which illustrates that only amylose constituents were esterified to potato starch. The ER effect of both phosphate and cellulose could not be found. On the other hand, ER fluid containing a particle volume fraction of 10% exhibits shear-thinning behavior even in the absence of an electric field [117]. The shear stress exhibits a plateau over a large range of shear rates. The plateau region of CP is much broader than that of PSP, and these regions become broader with increasing phosphoric acid concentration.

To better understand the ER performance of the cellulose based ER fluids, their dielectric properties were measured, and then correlated with the ER characteristics of their rheological properties.

**Figure 13:**
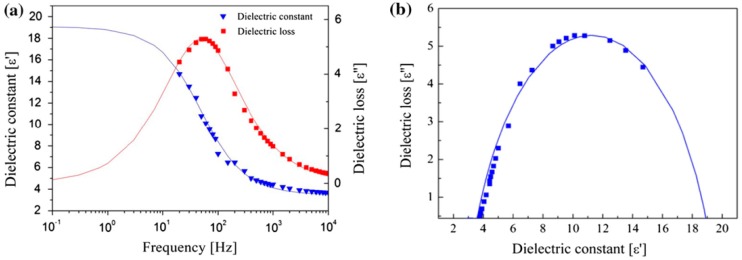
Dielectric properties of an MCC particle based ER fluid: (**a**) dielectric constant and loss factor vs. frequency; and (**b**) dielectric loss vs. dielectric constant (Cole–Cole plot) [93].

The dielectric spectra of the MCC particle based ER fluid were measured using a LCR meter, as shown in Figure 13. The dielectric constant (ε′) and dielectric loss (ε″) are known to play a significant role in polarizability, which is important for the ER effect of ER fluids. Figure 13 shows ε′ and ε′′ as functions of frequency in the range from 0.1 to 10,000 Hz. The solid lines were fitted with the Cole–Cole equation, given below as Equation (3):

(3) $$\varepsilon^{*}=\varepsilon^{\prime }+i\varepsilon^{\prime \prime }=\varepsilon_{\infty }^{\prime }+\frac{\varepsilon_{0}^{\prime }-\varepsilon_{\infty }^{\prime }}{1+{\left(i\omega \lambda \right)}^{1-\alpha }}(0\leq \alpha <1)$$

Here, ε_0_ is the relative permittivity when the frequency is near 0, and ε_∞_ is the dielectric value when the frequency is infinite [111]. The difference between these values, ∆ε = ε_0_ − ε_∞_, represents the polarizability of the ER fluids, which is related to the electrostatic interactions between the conducting particles. The index (1−α) represents the broadness of the relaxation time distribution. The relaxation time of λ = 1/2πƒ_max_ is the speed of interfacial polarization in the presence of an electric field, in which the ƒ_max_ represents the maximum ε″. In this study of the MCC based ER fluid, the values of the parameters of ε_0_, ε_∞_, α, and λ were 19.1, 3.52, 0.245, and 0.0032, respectively, revealing a very rapid polarization response of the MCC conducting particles under an applied electric field.

## 7. Conclusions

This study provides a concise review of the preparation of several kinds of cellulose particles and cellulose composites, based on cellulose obtained from different source materials and manufacturing processes, as well as the behavior of electric stimuli-responsive ER fluids based on these particles under external electric fields. The measured ER characteristics show that the cellulose based ER fluids exhibit typical ER behaviors. At zero electric field, the ER fluids retain a liquid-like state, showing Newtonian fluid properties; however, once the electric field strength is increased, the particles immediately form fibrillar structures, causing the ER fluid to transition to a solid-like state due to the attractive forces between the dipole moments of the dispersed conductive particles. The flow curves of shear stress were better fitted using the CCJ model as compared to the Bingham model. The dependence of their yield stresses on the applied electric field strength was also correlated using a universal yield stress equation. Compared to hydrous pristine cellulose based ER fluids, anhydrous cellulose derivatives such as phosphate cellulose and cellulose carbamate were found to demonstrate better ER performance. Furthermore, rod-like shape of these particles could be advantageous for improving ER efficiency. Hence, the cellulose materials were found to possess important potential for smart ER fluids in industrial applications, among various potential candidate materials in terms of abundance with low cost, eco-efficiency and typical ER characteristics.

## Figures and Tables

**Table 1:** Chemical composition of cellulose containing materials (Adapted from [29]).

Type of Biofiber	Composition (%)				
	Source	Cellulose	Hemicellulose	Lignin	Extract
Wood	Hardwood	43–47	25–35	16–24	2–8
	Softwood	40–44	25–29	25–31	1–5
Non-wood	Bagasse	40	30	20	10
	Coir	32–43	10–20	43–49	4
	Corn cobs	45	35	15	5
	Corn stalks	35	25	35	5
	Cotton	95	2	1	0.4
	EFB	50	30	17	3
	Flax(retted)	71	21	17	3
	Flax(unretted)	63	12	3	13
	Hemp	70	22	6	2
	Henequen	78	4–8	13	4
	Istle	73	4–8	17	2
	Jute	71	14	13	2
	Kenaf	36	21	18	2
	Ramie	76	17	1	6
	Sisal	73	14	11	2
	Sunn	80	10	6	3
	Wheat straw	30	50	15	5

**Table 2:** Optimized values of parameters for the Bingham model for the MCC ER fluid is Figure 6a (reproduced from [93]).

Parameters	Electric Field Strength (kV∙mm^−1^)				
	0.3	0.5	0.7	1.0	1.3
τ_y_	23.5	52.9	96.5	175	272
η_0_	0.08	0.06	0.03	0.01	0.001
